# Supplementary figures and images for: Immunotherapeutic potential of collagen V oral administration in mBSA/CFA-induced arthritis
Source: PLoS One. 2024 Oct 8;19(10):e0311263. doi: 10.1371/journal.pone.0311263 (PMC11460680; doi:10.1371/journal.pone.0311263)

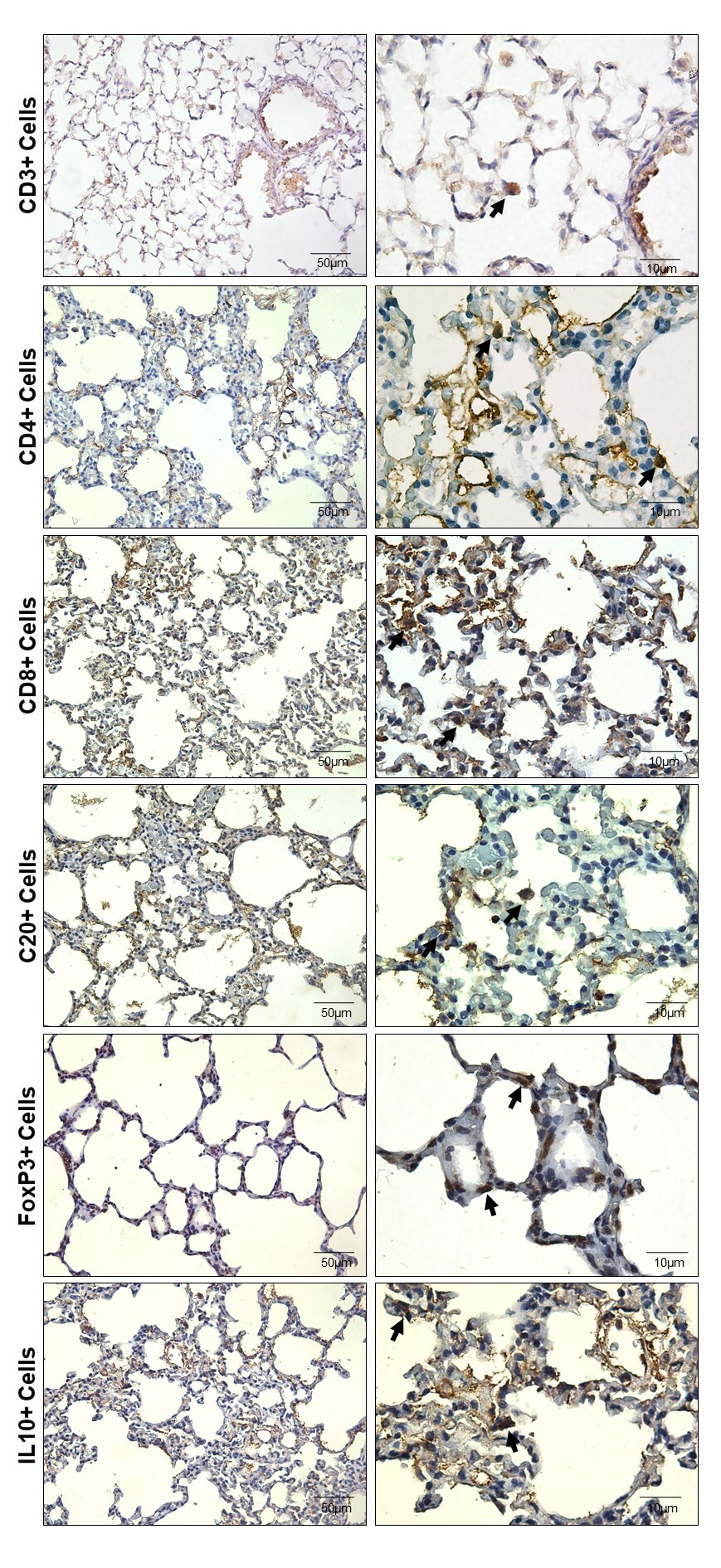

Supplement: S1 Fig — Immunostaining for CD3, CD4, CD8, CD20, FoxP3, and IL-10 (arrows) was performed on normal rat pulmonary tissue. Original magnifications: 400x left panel and 1000x right panel. (TIF) [file pone.0311263.s001.tif]

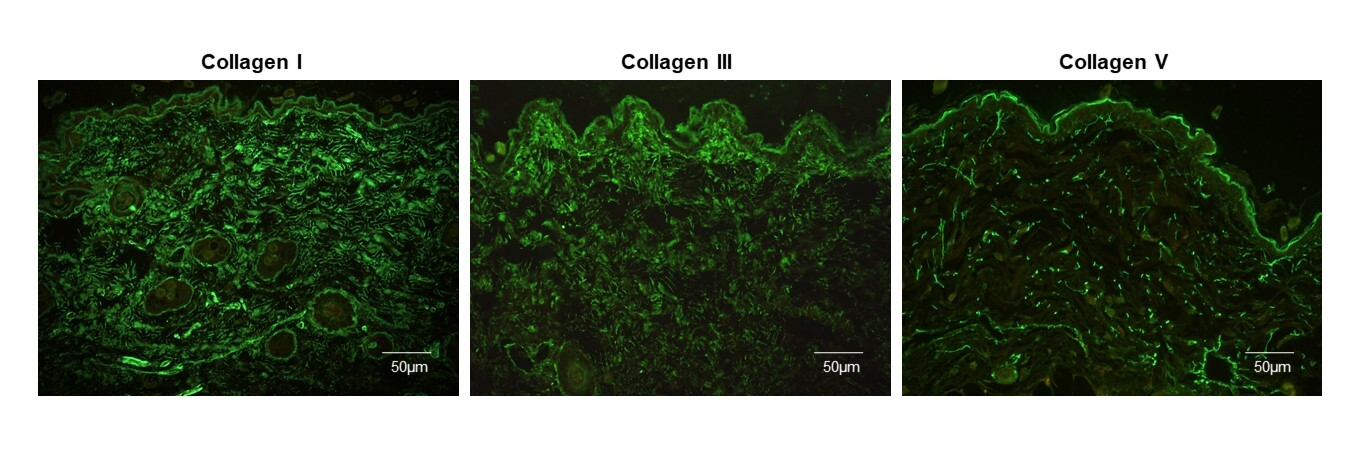

Supplement: S2 Fig — Immunostaining for Col I, III and V was performed on normal rat skin. Original magnification: 400x. (TIF) [file pone.0311263.s002.tif]

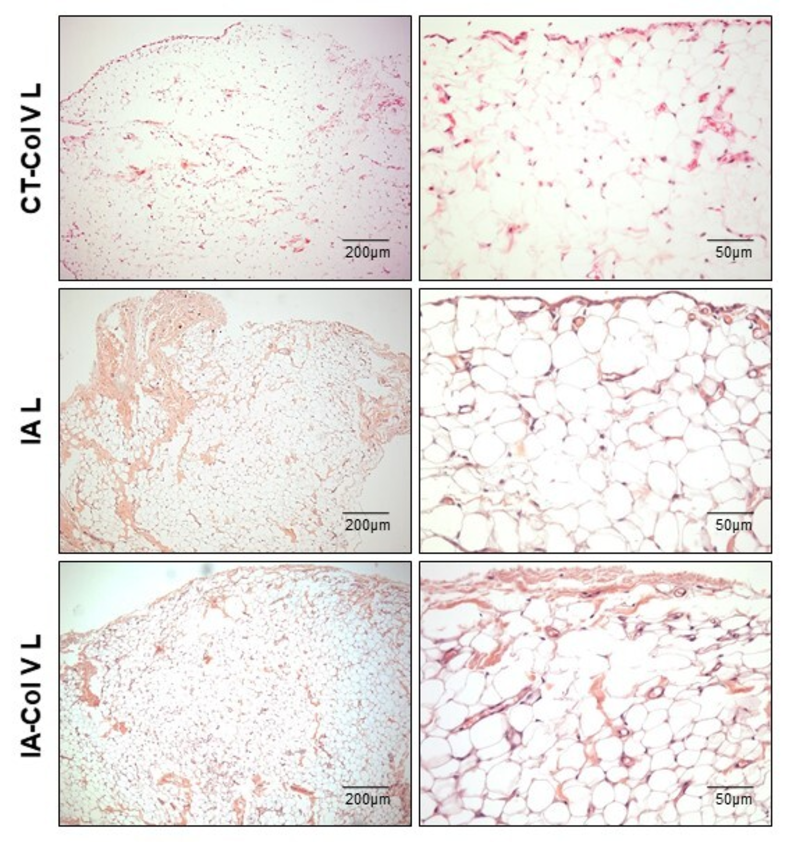

Supplement: S3 Fig — H&E-stained synovium from the left (L) joint of the CT-Col V, IA, and IA-Col V groups. Original magnification: 40x left panel and 400x right panel. (TIF) [file pone.0311263.s003.tif]
