# Supplementary material for: Immunotherapeutic potential of collagen V oral administration in mBSA/CFA-induced arthritis
Source: PLoS One. 2024 Oct 8;19(10):e0311263. doi: 10.1371/journal.pone.0311263 (PMC11460680; doi:10.1371/journal.pone.0311263)
Supplement: S1 Dataset — Mean of each analysis performed in IA and IA-Col V groups. (PDF) [file pone.0311263.s004.pdf]

Krenn's histopathological score for evaluation of the degree of synovitis of IA and IA-Col V groups

Score			
IA R	IA L	IA-Col V R	IA-Col V L
9	1	1	0
5	0	4	0
9	1	2	1
5	0	3	1
5	0	2	0
5	0	3	0
9	2	0	2
6	0	2	0
5	0	3	2
7		2	

Quantification of [18F]FDG uptake presented as the standardized uptake value (SUV) of the right (R) and left (L) knees of IA and IA-Col V groups

PET/CT										
	IA R									
Basaline	0.57	0.53	0.47	0.64	0.58	0.51	0.41			
14 days	0.98	0.77	0.89	0.58	0.7	0.93	0.6			
30 days	0.78	0.65	0.82	0.67	0.61	0.81	0.53			
	IA L									
Basaline	0.51	0.41	0.61	0.53	0.61	0.5	0.42			
14 days	0.61	0.51	0.47	0.31	0.41	0.4	0.31			
30 days	0.43	0.5	0.48	0.59	0.33	0.33	0.38			
	IA-Col V R									
Basaline	0.59	0.55	0.45	0.54	0.7	0.58	0.42	0.55	0.37	0.29
14 days	0.9	0.82	0.8	0.95	1.09	0.99	1.09	0.42	0.54	0.46
30 days	1.04	0.81	0.48	0.79	0.89	1.09	1.05	0.43	0.61	0.49
	IA-Col V L									
Basaline	0.44	0.51	0.39	0.44	0.58	0.6	0.49	0.52	0.4	0.29
14 days	0.62	0.64	0.61	0.64	0.72	0.68	0.74	0.41	0.35	0.35
30 days	0.78	0.58	0.36	0.72	0.71	0.66	0.77	0.43	0.44	0.45

Quantification of CD3+, CD4+, CD8+, CD20+ and CD68+ cell expression in synovial tissue of IA and IA-Col V groups

CD3+			
IA R	IA L	IA-Col V R	IA-Col V L
62.08	6.17	46.68	0
83.99	0	42.43	4.17
52.83	7.35	32.49	6.37
63.3	0	51.97	3.61
80.03	0	50.61	2.68
60.82	0	37.19	8.25
41.81		59.41	1
53.82			5.13
			2.11
			3.36
			3.53

CD4+			
IA R	IA L	IA-Col V R	IA-Col V L
41.84	2.86	9.85	0
61.71	9.76	9.85	5.6
53.05	0.91	31	6.99
53.17	6.52	30.85	1.75
23.21	9.02	27.35	8.43
21.42	7.75	28.07	0.77
24.76	0.57	29.01	6.47
	1.39	31.48	3.68
		9.65	4.17
		23.1	2.08

CD8+			
IA R	IA L	IA-Col V R	IA-Col V L
44.48	0	42.55	1.43
68.73	8.1	49	4.36
74.71	5.41	45.93	0
40.75	2.81	23.85	5.64
51.61	15.97	51.86	0
46.97	10.04	50.21	3.72
44.85	10.25	52.61	6.92
	0		0
			0

CD20+			
IA R	IA L	IA-Col V R	IA-Col V L
46.83	1.11	28.62	5.36
38.77	2.87	11.02	8.37
82.47	2.98	45.16	6
84.78	4.69	44.65	0
39.88	2	17.03	8.47
31.46	4.36	46.01	5.66
51.03	3.94	34.86	7.17
	0	25.09	3.61

CD68+			
IA R	IA L	IA-Col V R	IA-Col V L
7.05	4.4	8.6	0
13.77	0	3.63	2.22
8.73	0	9.39	0
8.8	0	6.76	0
7.96	0	8.71	0
19.2	0	2.97	2
		4.92	
		4.88	
		3.12	
		4.93	

Anti-collagen II and V antibodies frequency in the sera of the control, IA and IA-Col V groups

Anti-collagen II antibodies		
Control	IA	IA-Col V
0.027	0.406	0.494
0.014	0.677	0.468
0.031	0.218	0.591
0.042	0.168	0.457
0.046	0.153	0.384
0.029	0.215	0.342
0.019	0.108	0.165
0.018	0.075	0.089
0.03	0.175	0.153
0.033	0.395	0.143

Anti-collagen V antibodies		
Control	IA	IA-Col V
0.041	0.142	0.166
0.043	0.403	0.251
0.023	0.213	0.263
0.023	0.189	0.273
0.043	0.201	0.282
0.029	0.378	0.157
0.036	0.23	0.249
0.031	0.255	0.32
0.051	0.189	0.302
0.04	0.14	0.209

Quantification of IL-10+ and FoxP3+ cell expression of IA and IA-Col V groups

IL-10+			
IA R	IA L	IA-Col V R	IA-Col V L
39.69	1.43	63.95	2.59
30.77	7.83	67.32	0.91
31.14	3.99	58.03	7.01
65.08	4.43	65.31	0.91
62.93	10.28	51.87	0
44.51	1.5	57.61	1.94
12.88	3.3	48.47	6.96
42.93	1.38	59.32	0
		66.53	0.83
		55.09	0

FoxP3+			
IA R	IA L	IA-Col V R	IA-Col V L
15.34	0	15.32	3.43
17.49	0	29.46	0
4.97	2.78	35.93	0
7.78	2.87	20.01	1
8.91	0	20.82	3.65
19.14	2.08	22.16	0.83
9.35	3.23	23.48	4.84
	6.19	19.38	5.85
			5.5
			5.6

Quantification of types I, III and V collagen expression of IA and IA-Col V groups

Collagen I			
IA R	IA L	IA-Col V R	IA-Col V L
7.67	15.97	15.61	8.41
11.65	7.18	21	7.21
12.96	16.17	16.98	8.59
17.46	14.19	18.67	10.56
12.53	8.96	16.01	16.37
10.15	15.38	17.46	11.81
8.93	6.02	23.87	14.43
12.15	9.92	15.3	8.88
6.84	7	14.45	15.26
		11.95	6.9

Collagen III			
IA R	IA L	IA-Col V R	IA-Col V L
4.57	6.32	4.24	5
2.13	3.45	6.75	5.44
2.96	4.28	5.56	4.2
4.09	4.93	4.28	3.81
5.54	4.56	3.93	4.71
1.24	1.85	2.17	2.58
1.13	1.24	1.99	2.2
1.22	1.21	1.93	1.66
	1.32	3.26	2.37
	2.19	1.22	3.37

Collagen V			
IA R	IA L	IA-Col V R	IA-Col V L
19.22	2.45	2.35	0.65
5.18	3.73	1.88	1.24
12.7	4.27	5.02	1.43
16.76	6.61	2.95	1.44
8.47	5.96	2.24	2.39
17.33	3.64	1.71	4.87
5.45	6.69	5.03	0.67
	4.93	2.1	0.62
	3.37	2.62	0.46
		2.89	5.59
